# CAR Talk: How Cancer-Specific CAR T Cells Can Instruct How to Build CAR T Cells to Cure HIV

**DOI:** 10.3389/fimmu.2019.02310

**Published:** 2019-09-27

**Authors:** Gloria B. Kim, Kristen Hege, James L. Riley

**Affiliations:** ^1^Department of Microbiology, Center for Cellular Immunotherapies, Perelman School of Medicine, University of Pennsylvania, Philadelphia, PA, United States; ^2^Celgene Corporation, San Francisco, CA, United States

**Keywords:** T cell, lentiviral (LV) vector, immune escape and surveillance, clinical trials, immune privilege

## Abstract

Re-directing T cells via chimeric antigen receptors (CARs) was first tested in HIV-infected individuals with limited success, but these pioneering studies laid the groundwork for the clinically successful CD19 CARs that were recently FDA approved. Now there is great interest in revisiting the concept of using CAR-expressing T cells as part of a strategy to cure HIV. Many lessons have been learned on how to best engineer T cells to cure cancer, but not all of these lessons apply when developing CARs to treat and cure HIV. This mini review will focus on how early CAR T cell studies in HIV paved the way for cancer CAR T cell therapy and how progress in cancer CAR therapy has and will continue to be instructive for the development of HIV CAR T cell therapy. Additionally, the unique challenges that must be overcome to develop a successful HIV CAR T cell therapy will be highlighted.

## How Initial HIV Studies Paved the way for Successful CD19-Directed CAR Therapy

From a T cell perspective, controlling HIV replication and cancer growth share many of the same challenges: antigen escape, antigen persistence resulting in T cell exhaustion, and active mechanisms employed by both HIV and tumors to avoid T cell recognition and elimination. Thus, the use of CARs to redirect T cells toward both HIV and cancer as a means to bolster T cell control of these maladies was an attractive concept, which led to the preclinical studies using both HIV and cancer models. In the 1990s when antiretroviral therapy (ART) was in its infancy and not yet able to provide durable control of HIV replication, the rationale to treat HIV infection with CAR T cell therapy advanced more rapidly, and in this setting, the first CAR T cell trials were performed. These studies tested the ability of T cells expressing a major histocompatibility complex (MHC)-unrestricted chimeric receptor consisting of CD4, as the natural ligand of the HIV Envelope (Env) glycoprotein, and the CD3 zeta (ζ) chain (1) to suppress viral replication in HIV–infected individuals (2–4). While clinical success was not achieved with these early efforts in the just-emerging CAR T cell field, these efforts were not a “failure,” but in fact, successfully laid fundamental groundwork that enabled success using CAR T cells to treat CD19-expressing tumors. Several key observations and discoveries foundational to the overall field of CAR T cell therapy were made during the clinical investigation of CD4-ζ CAR T cells. For one, the field gained an appreciation that a combination of CAR-modified CD4 and CD8 T cells, rather than purified CD8 T cells alone, resulted in a marked improvement in CAR T cell persistence (3). This was ultimately confirmed by demonstration of >10 years of durable CD4-ζ CAR T cell detection in treated subjects (5). Additionally, these early studies demonstrated that rapid and reproducible CAR T cell manufacturing could be achieved both from uninfected and viremic HIV-infected subjects following 10-day culture incorporating T cell co-stimulation with anti-CD3 and anti-CD28 immuno-magnetic beads. This manufacturing process resulted in improved functional properties of CD4-ζ CAR T cells as well as stable and durable *in vivo* persistence (3–5). Moreover, evidence in randomized trials suggested modest anti-viral activity in HIV-infected subjects through demonstration of trends in reduction of blood- and gut-associated HIV reservoirs, and a reduction in transient viral rebound in plasma (or “blips”) in aviremic subjects (2, 4). Finally, these studies demonstrated a lack of immunogenicity of the fully human CD4-ζ construct and an absence of depletion of MHC class II expressing cells, suggesting that CD4-MHC class II interaction was not sufficient to trigger CAR activity. Of note, these early trials with CD4-ζ CAR T cells were performed with the first generation CAR constructs using gamma-retroviral vectors and including only the CD3-ζ cytoplasmic domain without the benefit of co-stimulatory molecules, such as CD28 or 4-1BB, included in successful modern CAR T cell trials. Additionally, these early HIV-specific CAR T cells were not protected from HIV infection, a risk that is further exacerbate by using CD4 as a retargeting domain. Recently, a CD4-based CAR that was re-engineered (see details below) to incorporate lessons learned from successful cancer targeting CARs (6), was shown to confer greater antiviral activity than widely-investigated broadly neutralizing antibody (BNAb) based CARs. This CAR coupled with agents to protect the CAR from HIV infection (7–10) has recently entered the clinic (NCT03617198) to determine whether these changes augment HIV CAR T cell activity and provide some durable control of HIV replication and/or reduce the latent reservoir. The evolution of CAR design is summarized in Table 1.

**Table 1:** Evolution of CARs used in HIV and cancer cell and gene therapy.

**Component**	**First generation HIV CARs (11)**	**CD19 CARs that led to first FDA approval (12)**	**Current HIV CARs being tested in NCT03617198 (6)**	**Functional impact**
Viral vector	γ Retrovirus (MMLV-based)	Lentivirus (HIV-based)	Lentivirus (HIV-based)	Safety, sustained expression
Promoter	PGK	EF1α	EF1α	Higher expression (MFI), sustained expression
Hinge	None	CD8α	CD8α	Flexibility
Transmembrane	CD4	CD8α	CD8α	Helps prevent infection, dimerization to promote activation
Signaling motifs	CD3ζ	CD3ζ, 4-1BB	CD3ζ, 4-1BB	Improved *in vivo* expansion, survival, and persistence
Extracellular domain	CD4 EC domains	scFv domains	CD4 EC domains	No immunogenicity or off target recognition. HIV's ability to escape will likely be limited

## Cancer and HIV: Shared Challenges and Opportunities

### Persistent Antigen and Exhaustion

Persistence of antigen at high levels drives exhaustion of T cells, which limits the functional properties of T cells and is characterized by high expression of immune checkpoint (IC) molecules, such as programmed death-1 (PD-1), and cytotoxic T-lymphocyte-associated antigen 4 (CTLA-4), ultimately hindering clearance of tumors and chronic infections (13–16). An advantage of CAR T cell therapy is that new, fully functional T cells can be redirected toward HIV or tumor antigens. Once re-infused, however, these CAR T cells are susceptible to becoming exhausted if they are unable to clear the targeted antigen in a timely manner. Thus, the reversal or prevention of T cell exhaustion may represent a mechanism whereby dysregulated immunity is prevented, allowing CAR T cells to have a longer therapeutic window to control either HIV replication or tumor cell growth.

Antibodies targeting ICs (e.g., PD-1, PD-L1 or programmed death-ligand 1, and CTLA-4) have shown clinical responses in multiple tumor types, including melanoma, renal cell carcinoma, non-small cell lung cancer (17), and bladder cancer (18). So far, there are six U.S. FDA-approved immune checkpoint inhibitors (ipilimumab, nivolumab, pembrolizumab, avelumab, atezolizumab, and durvalumab) and their objective response rates have ranged from 27% in melanoma patients, to 30% in non-small cell lung cancer patients, and 63% in Kaposi sarcoma patients (19). However, there have been significant immune-related toxicities, including onset of type 1 diabetes, colitis, and dermatological issues (20) that may represent an acceptable risk/benefit to advanced cancer patients, but may be unacceptable to HIV-infected individuals whose viral load is well-controlled by ART. Several clinical trials are currently underway to explore the effect of anti-PD-1 based therapies in HIV-infected individuals who also have tumors known to be responsive to PD-1 blockade (NCT03367754, NCT02408861) (19) and one trial is treating non-tumor bearing HIV-infected individuals (NCT03787095). It will be interesting to see if and, if so to what extent, anti- PD-1 therapies can re-invigorate the HIV-1 specific immune response and whether side effects of this anti-PD-1 therapy in this otherwise healthy population confer an overall benefit/risk sufficient to permit wider exploration in HIV Cure studies.

Furthermore, some studies show that PD-1 also contributes to the establishment and maintenance of HIV latency, so checkpoint blockade may be a promising approach to reverse latency (21). In order for the remaining hidden pool of virus to become recognized by HIV–specific T cells, it must be reactivated first and this could be accomplished by various latency reversing agents (LRAs) (e.g., histone deacetylase inhibitors (HDACis) and protein kinase C class drugs) (22). IC blockades could also function to reverse HIV latency through limiting inhibitory signals sent from IC molecules into cells harboring latent HIV. CTLA-4 blockade results in significant increases in plasma viremia and T cell activation (23). Thus, the combination of IC blockade coupled with HIV CAR T cell therapy may be an effective “shock and kill” (24) strategy.

If systemic checkpoint inhibitor approaches prove too toxic for routine use in HIV-infected individuals, specific targeting of checkpoint genes within HIV-specific CAR T cells via clustered regularly interspaced short palindromic repeats (CRISPR) or small hairpin RNA (shRNA) technologies may prove an effective and safe way to make HIV-specific CAR T cells exhaustion resistant because only the HIV CAR T cells will have their checkpoint genes disabled (25, 26). Here, cancer-based therapies are paving the way for HIV-specific therapies. A clinical trial using a CRISPR-based approach to disable PD-1 is currently underway (NCT03399448) to determine if this improves the anti-tumor efficacy of engineered New York esophageal squamous cell carcinoma 1 (NY-ESO-1), a cancer-testis antigen expressed in a wide range of tumor types -targeted T cells. If successful, this trial could establish sufficient safety and feasibility to warrant coupling HIV CARs with PD-1 CRISPRs. Other immune checkpoint inhibitors, such as those targeting T-cell immunoglobulin and mucin-domain containing-3 (Tim-3), lymphocyte-activation gene 3 (LAG-3), and T-cell immunoreceptor with Ig and ITIM domains (TIGIT), may also help enhance anti-HIV CAR T cell therapy by overcoming T cell exhaustion, possibly with a more acceptable safety profile (27–30).

### Antigen Escape

Antigen escape and efforts to limit T cell recognition of targeted cells are major hurdles for effective T cell-based HIV and cancer control (13). Most common mechanisms of antigen escape in cancers are (1) the immune selection of cancer cells, which lack or mutate immunogenic tumor antigens or lose expression of the antigens targeted by CAR T cells, (2) the acquisition of defects or deficiencies in antigen presentation [e.g., loss of major histocompatibility (MHC) expression], or (3) deficits of antigen processing machinery (31–33). Multiple compelling studies suggest that aberrant signal transducer and activator of transcription 3 (STAT3) signaling plays a key role in facilitating tumor escape from immune detection by impairing antigen presentation and reducing production of immunostimulatory molecules (34). Thus, STAT3 inhibition in concert with other immunostimulatory agents, such as toll-like receptor (TLR) 3, TLR7, and TLR8 agonists like stimulator of interferon genes (STING) or retinoic acid inducible gene (RIG)-I, could provide promising combination immunotherapeutic strategies. Additionally, a variety of CD19 mutations and alternative splicing have been observed with development of acquired resistance of acute lymphocytic leukemia (ALL) to CD19 targeted CAR T cells (35). In this regard, CARs targeting distinct motifs on the tumor surface may be an effective strategy to prevent resistance through tumor escape. For example, Ruella et al. demonstrated that the combination of CD123-targeted and CD19-targeted CAR T cells prevented relapses caused by antigen loss in preclinical models (36). Another study used bispecific CARs that targeted both CD19 and CD20 in order to minimize antigen escape from CD19-negative leukemia. Those bispecific CAR T cells were able to eradicate heterogeneous populations of leukemic cells in NSG mice (37).

In the case of HIV, the virus has evolved features to escape from immune monitoring with quick selection for cytotoxic T lymphocytes (CTL) escape mutations prior to antiretroviral therapy (ART) due to an error prone reverse transcriptase (10). Additionally, the HIV-1 negative regulatory factor (Nef) protein modulates expression of MHC class I, CD28, and other proteins involved in immune recognition to evade CTLs (38–40). As a result, recent efforts have focused on introducing a potent engineered immune response designed to overcome HIV's escape mechanisms instead of solely relying on the endogenous immune response to control HIV replication in the absence of ART (41–43). One advantage of CARs to target HIV is that HIV Env expression on the cell surface is not affected by Nef; thus, CAR T cells may recognize HIV-infected cells better than natural HIV-specific T cells. HIV can rapidly escape from a single BNAb (44–46), and will likely escape from a CAR that uses a BNAb as its targeting domain, though those targeting the CD4 binding site seem to be more resistant to escape (47). However, like in cancer, bi- or multi-specific HIV CARs have been constructed and have demonstrated superior efficacy against several HIV-1 primary isolates *in vitro*, warranting further *in vivo* investigation (8, 10, 48, 49). Moreover, it is not clear whether use of BNAb is advantageous as a means to redirect T cells to HIV as BNAb binding relative to non-BNAb binding promotes Env internalization (50). Thus, in both HIV and cancer, loss of target recognition by CAR T cells via antigen escape is an issue, but through simultaneous targeting of multiple antigens or the targeting of biologically important functions such as HIV binding to CD4, this issue seems to be solvable.

### Immune-Privileged Sites

Immune privileged sites are anatomical regions (CNS, testes, and eyes) in which the immune response is purposely attenuated, usually to protect sensitive tissue from immune-related, off-target damage. These immune sanctuaries are often used by HIV and some tumors to hide from the immune attack. To overcome these issues, recent preclinical studies have shown the antitumor efficacy and safety of intracranial administration of EGFRvIII, HER2, and IL13Rα2 redirected CAR T/NK cells. Brown et al. described a patient who received multiple infusions of IL13Rα2-CAR T cells over 220 days via infusions to the resected tumor cavity and the ventricular system (51, 52).

Immune privilege coupled with HIV latency is an even more daunting problem for T cell-based therapies targeting HIV. Recent data have highlighted the fact that the >99% of all HIV-infected CD4+ T cells are found outside the vasculature within secondary lymphoid organs (SLOs), gut, brain, lung, and other tissues (53). Immunologic clearance of these infected cells is thought to largely involve cytotoxic CD8+ T cells, specifically CD8+ T cells with a fully differentiated “CTL” phenotype (CCR7-CD62L-CD27-CD45RA+) (54–59). CTLs, however, do not bear the markers (CCR7 and CD62L) necessary to enter lymphoid tissue (60–63). Betts and colleagues recently demonstrated that peripheral blood CTLs are rarely found in HIV-infected lymph nodes, and instead lymph nodes are populated by HIV-specific CD8+ T cells with very limited cytotoxic function (64, 65). In addition, it has been demonstrated that intestinal mucosal tissue is similarly populated with CD8+ T cells that have limited cytotoxic function (66). HIV-infected CD4+ T follicular helper cells (T_FH_ cells) in B cell follicles of lymphoid tissue are a major compartment for persistent virus replication during combination ART (cART) (67–69). Even though virus-specific CTLs have been detected in lymph nodes, they are largely absent from the B cell follicles because they lack expression of CXC-chemokine receptor 5 (CXCR5), which is responsible for the trafficking of cells into the B cell zone along a CXC-chemokine ligand 13 (CXCL13) concentration gradient (70, 71). Therefore, the lack of CXCR5 expression on virus-specific CTLs is one mechanism that promotes the persistence of infected CD4+ T_FH_ cells within an immune-privileged site (72). On the other hand, increasing evidence suggests the existence of tissue-resident macrophages as HIV-1 reservoirs (73, 74). Allers et al. found that macrophages were significantly enriched in the gut of untreated HIV patients (75). This also corresponds with a decrease in blood monocytes and increased expression of gut homing receptors (e.g., chemokine receptor CCR9 and integrin α4β7) on those monocytes, suggesting that blood monocytes may be a major source of macrophages that infiltrate gut mucosa. It has been reported that α4β7 is able to bind HIV-1 Env protein gp120 and is 3-fold larger than CD4 receptor, allowing it to capture HIV efficiently (76). Lastly, it is unclear whether engineered T cells will be able to transverse the blood brain barrier in HIV-infected individuals in order to target the HIV reservoir hiding in the CNS (77).

Taken together, there are at least three major issues facing HIV CAR T cells: (1) Will the latent reservoir of HIV-infected cells express sufficient levels of the target antigen (e.g., HIV Env) to drive CAR T cell recognition after a latency reversal agent is used? (2) Will the HIV CAR T cell be able to traffic to the site where the HIV-infected cell is hiding? and (3) if it is expressing antigen and the HIV CAR T cell is able to recognize the infected cell, will the CAR T cell have the necessary machinery (perforin and granzyme) that may be lost as part of the T cell exhaustion program to kill the HIV-infected cell and eliminate the latent reservoir?

## Cancer and HIV: Unique Challenges and Opportunities

### Cancer CAR T Cells Are Infused When Antigen Level Is High; HIV CAR T Cells Are Infused When Antigen Level Is Low

Unless employed to prevent tumor relapse or treat minimal residual disease, cancer-specific CAR T cells are generally infused when there is abundant target antigen available. CAR T cells that quickly recognize their target have an engraftment advantage (78, 79). Moreover, CAR T cell recognition and killing of target cells can result in massive expansion of CAR T cells. In one celebrated case, a single CAR T cell whose vector integrated into and disrupted the function of the Tet methylcytosine dioxygenase 2 (Tet2) gene preferentially expanded to >90% of all of the CAR T cells within the body and this clone was able to maintain durable control of the targeted leukemia (80), indicating that CAR T cells have massive expansion potential. Thus, for individuals with established tumors, it may be possible to infuse a small number of well-engineered T cells and let the body serve as the bioreactor to generate enough T cells to eradicate the targeted tumor. However, CAR T cells that enter a body without significant target antigen may massively contract with a small subset becoming memory T cells, similar to what happens in a natural T cell response once antigen is cleared. Initial studies (NCT03617198) propose to infuse HIV CAR T cells in individuals whose ART has fully suppressed viral replication. It is unclear how well these adoptively transferred T cells will engraft in the absence of high levels of target antigen; however, it is reassuring that first generation CAR T cells targeting CD4-ζ demonstrated brisk expansion and prolonged persistence following infusion into aviremic patients effectively managed with ART therapy (2). For approaches that attempt to block viral rebound once ART is removed, there needs to be a sufficient quantity of T cells present that are widely distributed throughout the body to recognize the vast majority of cells expressing HIV Env as soon as they emerge. Thus, strategies such as infusion of very high numbers of T cells or vaccination approaches that maintain high levels of HIV-specific CAR T cells in the presence of minimal antigen may be required for HIV-specific CAR T cells to be used as part an HIV cure strategy (68).

### HIV Can Be Specifically Targeted, but HIV Can Target the CAR T Cells

The search for a CAR target that uniquely recognizes a tumor has proven very challenging. Currently, targets fall into two categories: (1) those with acceptable on-target/off-tumor toxicity, i.e., loss of “expendable” tissue such as B cells in the case of CART-19 therapies or (2) targets highly expressed on tumors and weakly expressed on a limited set of healthy cells, which may allow the CAR T cells to preferentially kill tumor with minimal effects on healthy cells. On-target/off-tumor recognition of CAR T cells has been observed in a variety of organ systems, including gastrointestinal, hematologic, and pulmonary (81). A fatal example of on target/off tumor CAR T cell recognition was observed with the cancer-associated antigen HER-2/neu. Rapid respiratory failure, multi-organ dysfunction, and subsequent death was attributed to reactivity against pulmonary tissue expression of HER-2/neu (82). Fortunately, for HIV CAR T cell therapy, HIV is non-self and thus highly specific agents can be developed that are unlikely to cross-react with human tissue. However, while HIV can be uniquely targeted, there are some challenges: (1) only the HIV Env protein is expressed on the cell surface after latency reversal, making it the only target suitable for CAR T cell therapy, and thus limiting some combinatorial approaches that may improve the efficacy and/or safety; (2) extensive sequence diversity within Env making it challenging to find antibody-based targeting agents that can bind all strains of HIV. Consequently, the natural HIV ligand, CD4, is attractive for use in a CAR construct, because HIV escape from binding to CD4, would likely result in a virus with greatly reduced fitness; (3) HIV Env expression levels are not fixed as in most cancer targets. Rather, the number of HIV Env targets on the cell surface increases over time as HIV replicates within the cell. However, the best chance for HIV CAR T cells to control HIV replication is to recognize and kill HIV-infected targets as soon as possible after infection when there is minimal HIV Env on the cell surface in order to limit the spread of the virus. Thus, CAR constructs that can redirect T cells to recognize minute levels of HIV Env on the cell surface will likely be very successful to limit HIV spread. This race between the CAR T cell to recognize HIV and HIV's effort to infect new cells has no clear parallel to cancer CAR T cells. It will therefore be interesting to see how this difference impacts the ability of HIV CAR T cells to control HIV replication in HIV-infected individuals.

Additionally, whereas tumors create hostile environments for T cells to function (83), HIV actively infects and kills T cells. While CD4 is a necessary binding receptor for most HIV strains, CD8 T cells can temporally express CD4 after T cell activation permits making both CD4 and CD8 HIV-specific CAR T cells susceptible to infection (6, 84, 85). For these reasons, HIV-specific CAR T cells will need to be protected from HIV infection. A variety of strategies exist including chemokine co-receptor disruption and fusion inhibitors that provide robust protection of T cells from infection (41). The only challenge in these strategies is the additional engineering that is required during the T cell manufacturing process.

### The Bar by Which Therapies Are Deemed Successful Differs Considerably Between HIV and Cancer Cell and Gene Therapy

Current cancer treatments such as chemotherapy, surgery, and/or radiation, have significant side effects and in most cases low rates of cure in advanced disease settings. CAR T therapies are currently being explored in patients with advanced/refractory malignancies and are FDA approved in chemotherapy refractory leukemia and lymphoma. Clinical success and FDA approval for Sipuleucel-T (Provenge), a dendritic cell-based therapeutic vaccine, was based on ~4 month increase in survival time for prostate cancer patients. In contrast, ART is nearly universally successful in compliant individuals with access to healthcare, and those individuals whose virus remains undetected due to ART have lifespans approaching those of non-HIV infected individuals (86). Thus, both commercial and clinical success for cancer therapies is measured by increasing mean survival time whereas for HIV, only a cure, whether functional or sterilizing (87, 88), is considered a success. Given that only two people have been cured of HIV infection (89, 90), having a lifetime cure as the only measure of success is quite a high bar. This is why analytical treatment interruptions (ATIs) are crucial to advance the HIV CAR T cell field. Here, individuals involved in an IRB approved clinical trial voluntary stop taking ART after receiving an experimental agent and the time to viral rebound is measured. Most individuals rebound within 2–4 weeks; therefore, individuals who are part of an interventional study that is able to limit the virus from replicating significantly longer provide evidence that the experimental therapy is having some effect. As the field matures and many approaches are studied, one can then analyze ATI data to propose combination trials to determine whether further delays in viral rebound occur. This combinatorial, iterative approach is likely the best chance we have to develop an effective and safe HIV Cure regimen. To date, carefully monitored ATIs have not resulted in ART escape or increased the viral reservoir (91–93), suggesting that there are no long term adverse outcomes for individuals participating in clinical trials that have ATIs (94).

## Outlook: Recent Lessons From Cancer Will Inform the Next Generation of HIV Specific–CARs

The development of cancer and HIV CAR T cell therapy has a long, intertwined, and symbiotic relationship (95), and this relationship is highlighted in Table 2. Exactly how did success with cancer CAR T cell therapy inform the design and implementation of HIV CAR T cell therapy? The initial CD4-ζ CAR was housed in a murine gammaretroviral vector, contained the CD4 transmembrane domain, lacked costimulatory domains, and was driven by the phosphoglycerate kinase (PGK) promoter (1, 112). In a side-by-side, step-by-step study, Leibman et al. compared this first generation HIV CAR with the vector design of CARs that achieved FDA approval for CD19-expressing tumors (6). Surprisingly, the choice of vector delivery made a huge difference in CAR expression and this translated into greater control of HIV replication. Substituting the EF-1a promoter resulted in both more stable and higher CD4 CAR expression. Replacing the CD4 transmembrane domain with the CD8 hinge region resulted in slightly less expression, but rendered the HIV CAR T cells less susceptible to infection and improved the overall efficacy of these T cells. Lastly, endowing the CD4 CAR with 4-1BB costimulation promoted both the survival and expansion *in vivo* as previously observed in tumor models (6, 113).

**Table 2:** Synergy between HIV and cancer cell and gene therapy.

**Advance**	**Initial impact**	**Impact on other disease**
Bone marrow transplant	Lifesaving approach to restore patient bone marrow after severe cancer therapy that can induce graph v. tumor effects (96)	Part of the regimen of the individuals cured of HIV (89, 90)
Retroviral vectors	The first time a genetically modified cell was infused into humans was when neomycin was expressed by a retroviral vector in cancer infiltrating T cells (97)	The clinical development of retroviral vectors in cancer paved the way for the first CAR T cell trial in HIV (3, 4)
CD3/28 bead culture system for T cell Stimulation	Development of a GMP compliant, robust method to expand HIV-infected CD4 T cells in the absence of ART due to CCR5 downregulation (98–101)	Used widely to manufacture T cells for cancer CAR therapy including in the first indication that led to FDA approval (12, 102–104) using SOPs initially developed for HIV
CAR T cell	Fusion of CD4 with the CD3 zeta chain created the first CAR construct tested in humans and demonstrated the long term persistence of CAR T cells (5)	Manufacturing advances and safety data obtained from HIV CAR T cell studies paved the way for development of the first FDA approval of any gene therapy- and the first CAR T product (12, 102, 105, 106)
Lentiviral vectors	A lentiviral vector that expressed anti-sense HIV Env in transduced T cells represented the first time lentiviral vectors were used in humans (107)	Lentiviral vectors have preferred integration pattern (108), improved expression (6), and are the preferred vector for cancer CAR T cell therapy
Genome editing	Infusion of CCR5 ZFN treated T cells into HIV-infected individuals represented the first time genome edited T cells were employed (109)	NYESO-1-specific T cells with disrupted TCR and PD-1 alleles were recently infused into cancer patients (NCT03399448)
TCR enhanced affinity	T cells expressing an affinity enhanced TCR specific for MAGE-A3 resulted in two treatment related deaths due to unexpected off-target toxicity (110, 111)	A clinical trial using similar technology to redirect T cells to HIV was stopped because the TCRs used did not undergo an improved screen for off target recognition (NCT00991224)

In a convergence of fields, much attention is now focused on where a CAR vector integrates. Pioneering studies by the Bushman lab demonstrated that HIV (and HIV-based vectors) prefers to integrate in coding regions, whereas murine gammaretroviruses target promoter regions (108, 114). More recently, the site of HIV integration has been shown to play a role in whether T cells will become part of the latent reservoir (115), suggesting that the site of integration can impact a T cell's long term persistence and ability to homeostatically expand. Using approaches to study how HIV integrates, Fraietta et al. uncovered how a CD19 CAR vector fortuitously integrated into the TET2 locus, and this integration resulted in a central memory-like T cell phenotype with an incredible ability to expand and function (116). As genome engineering becomes more effective, safer and less expensive (117), one can imagine that it will be possible to specifically insert a CAR vector into a precise spot in the genome to provide a functional advantage or survival benefit to either HIV or cancer CAR T cells.

As mentioned in the beginning, the field of T cell manufacturing was in its infancy when the first HIV CAR T cell therapy trials were performed. The field has matured considerably, but there is much more to learn in order to improve how T cells are produced for use in adoptive T cell applications. Cancer CAR T therapy has seen a strong correlation in how well T cells expand *ex vivo* with their *in vivo* function and persistence (118). Additionally, it has been demonstrated that changes in T cell manufacturing such as expanding T cells in the absence of human serum (119) improves the *in vivo* efficacy of CAR T cells. Here, developers of cancer CAR and HIV CAR can support each other as many of the developments in T cell manufacturing are likely to benefit both fields. One possible difference is that for HIV CAR T therapy large quantities of HIV CAR T cells may be required to have enough effectors on hand to prevent viral rebound after ART removal since there is minimal antigen present to induce *in vivo* CAR T cell expansion. In contrast, for cancer CAR T cell therapy, infusion of less CAR T cells may be safer, less expensive and just as effective so the manufacturing for these two therapies are reasonably similar now but they may diverge considerably once we learn more about what is required to obtain therapeutic responses. Lastly, HIV-infected individuals are currently excluded from receiving CAR T therapy in part because the commercial manufacturers have not developed a process by which HIV-infected T cells can be GMP manufactured. Perhaps one of the last gifts HIV CAR therapy can give to cancer CAR therapy is to share the best practices by which HIV CAR T cells are manufactured using T cells from HIV-infected individuals so that HIV-infected individuals can benefit from this life saving, cancer CAR T cell technology.

## Author Contributions

GK, KH, and JR co-wrote and edited the manuscript.

### Conflict of Interest

KH is an employee of Celgene and holds equity in Celgene, Mersana Therapeutics, and Arcus Biosciences. None of these companies have active HIV therapeutics programs. JR holds equity in Tmunity Therapeutics. The remaining author declares that the research was conducted in the absence of any commercial or financial relationships that could be construed as a potential conflict of interest.
